# Infant feeding and the media: the relationship between *Parents' Magazine *content and breastfeeding, 1972–2000

**DOI:** 10.1186/1746-4358-1-10

**Published:** 2006-04-30

**Authors:** Katherine A Foss, Brian G Southwell

**Affiliations:** 1 University of Minnesota, School of Journalism and Mass Communication, 111 Murphy Hall, 206 Church Street SE, Minneapolis, MN 55455, USA

## Abstract

Mass media content likely influences the decision of women to breastfeed their newborn children. Relatively few studies have empirically assessed such a hypothesis to date, however. Most work has tended to focus either on specific interventions or on broad general commentary about the role of media. In this study, we examined infant feeding advertisements in 87 issues of *Parents' Magazine*, a popular parenting magazine, from the years 1971 through 1999. We then used content analysis results to predict subsequent changes in levels of breastfeeding among U.S. women. When the frequency of hand feeding advertisements increased, the percentage change in breastfeeding rates reported the next year generally tended to decrease. These results underscore the need to acknowledge the potential role of popular media content in understanding breastfeeding patterns and public health trends.

## Background

We often assume that mass media content can affect health decision-making. A wide literature, in fact, documents numerous mass media campaigns in the past century that were predicated on that idea [1]. Actually detecting and documenting such media effects, however, has been a challenge less often met successfully. Moreover, some public health practitioners focus their attention on creating public service announcements and ignore the impact of other types of media content. There clearly is important work left to do in this arena.

Perhaps the most useful ideas as to how to locate and determine effects on health have arisen when researchers have stepped beyond the confines of a single, carefully-planned campaign evaluation to instead look at the impact of an array of media content on health beliefs and behavior [2-5]. For example, Southwell and colleagues looked at the relationship between news coverage and mammography seeking among U.S. women [5]. In that work, there does appear to be a striking relationship between the presence of supportive or dissuasive news content and the proportion of women who obtained cancer screening. In light of these results, can we detect a similar relationship between media content and breastfeeding?

As we will discuss, the proportion of women breastfeeding has varied over recent decades. These fluctuations are vitally important from a medical perspective, as individuals' decisions to breastfeed (or not) carry significant consequences for infant health. Studies have shown that artificially fed infants experience higher risks of illness and allergies, and, on average, require more extensive dental care than breastfed infants [6-9]. Also, breastfeeding creates an emotional bond between mother and child [10]. Considering these outcomes, it is important to understand what may influence mothers' decisions. Can trends in media content help to explain these vacillations? Before we answer that question, we first can look closer at recent patterns in the behavior.

Numerous studies on U. S. breastfeeding rates date back to at least the 1940s. Most research has centered on the fluctuation of breastfeeding rates in the twentieth century, illustrating how breastfeeding rates declined throughout much of the twentieth century, reached an all-time low in the early 1970s, and have tended to increase since that time [11-15].

Scholars have offered a number of explanations for the steep decline: increased hospitalization and anestheticization of new mothers, medical promotion of infant formula, and the use of feeding schedules all likely discouraged women from breastfeeding [16-18]. In addition to these factors, it is also probable that there is a cultural explanation worth considering, as the growing connection between infant formula and consumption and modernity – two important values, which discourage natural practices like breastfeeding, present in much mid- to late-20^th ^century U.S. media content, for example – also may have promoted infant formula usage [18-20].

American breastfeeding rates experienced their record low in 1971, when an estimated twenty-one percent of the population breastfed their infants at birth [14]. By this time, however, breastfeeding campaigns had started to develop. With the onset of the late 1960s and 1970s, some feminists and other advocates began campaigning for mothers to breastfeed their infants [17]. These campaigns, together with a changing ideology about infant feeding, may have helped to reverse existing trends. Over the next decade, in fact, breastfeeding rates rose dramatically. By 1984, nearly sixty percent of new mothers chose to breastfeed their children [14].

Contemporary studies suggest that the decision to breastfeed continues to be a complex issue, shaped by a multitude of factors, such as a mother's prenatal care and the influence of her health professionals, her perception of the father's views on breastfeeding, and her fear of lack of an adequate milk supply for the infant [17,21-23].

In the last thirty years, breastfeeding rates have generally increased, though there have been periods of slight decline. According to the 2001 Ross Laboratories Mothers Survey (RLMS), almost 70 percent of mother's initiated breastfeeding, which marks the highest breastfeeding initiation rate ever recorded in the United States [14,24]. Mothers also have been nursing for longer periods of time. Results from the RLMS indicated that almost a third of mothers were breastfeeding their infants six months after birth [14,24].

In sum, in past decades in the United States breastfeeding rates have fluctuated significantly. From the slow decline of breastfeeding in the early 1900s, the sharp descent of rates in the 1950s, and the growth of breastfeeding rates since this time, many external factors have been proposed as explanations for this variation, including changes in hospital resources and popular culture trends. Consideration of these various factors raises the question: what specifically might have been the role of exposure to media content?

For those who view reality as being socially constructed, media outlets can shape and reinforce dominant ideologies and convey these messages to a mass audience through systems of representation [25]. Under this assumption, it is likely that media messages about infant feeding influences how a mother decides to feed her infant.

While journalists, advocates, and others shaping media content have certainly promoted breastfeeding at times, especially in the 1920s and the late 1970s, many commentators have suggested that mass media, specifically advertising, actually has often discouraged breastfeeding, by diffusing information about infant formula products, reinforcing many of the factors that discouraged breastfeeding, and promoting modernity and social status. Marchand stresses the importance of media in the adoption of new technology, stating that "inventions and their technological applications made a dynamic impact only when the great mass of people learn of their benefits, integrated them into their lives, and came to lust for more new products" [19]. Through advertising, media not only alerts the public to new merchandise, but also teaches people why they need the product. By informing new parents of commercial milk substitutes and emphasizing their need for the product, media outlets likely encouraged the widespread adoption of breastfeeding alternatives.

Likewise, news organizations may have contributed to breastfeeding rate decline at times by perpetuating the myths that breastfeeding was dangerous. Often in the 20th century, a few cases of breastfeeding failures, such as infant starvation or the transmission of a mother's illness through her breast milk, would receive widespread publicity [18]. Although the negative consequences of bottle feeding dramatically outweighed breastfeeding from the perspective of health professionals, mothers may have believed that infant formula was less risky and therefore chose to bottle feed their infants [18].

The sexualization of the breast in the 1900s, in which mass media outlets played no small role, also may have been a factor in explaining periodic declines in breastfeeding. Early in the century, numerous creams, mechanical devices, and medicinal remedies were developed to supposedly "enhance" breast size and appearance [18]. Through advertising, women not only 'learned' of these specific products, but also may have learned that breast size and appearance were an important part of physical attractiveness [18]. With less emphasis on the breast as a utility, more women viewed breastfeeding as archaic or obscene [18].

Advertising also has tended to promote the idea of "scientific motherhood," particularly since the 1950s. Apple suggests that brochures and advertisements in the 1950s commonly promoted the modernity of infant formula and associated the use of scientific developments with quality parenting [20]. Many advertisements often conveyed the idea that a "good" mother does not simply rely on instincts but gathers knowledge about infant feeding and care [20]. Likewise, advertisements often promoted infant formula as an "elite" method of feeding one's infant, associating bottle feeding with higher class and modernity [17]. From these advertisements, mothers may have learned that if they did not use infant formula, society would consider them to be old-fashioned, uneducated, and perhaps of a lower class.

Contemporary research indicates that the topic of infant feeding is pervasive in media content around the world and yet is not discussed consistently. In a study of British media, Henderson and colleagues found numerous references to infant feeding on television and in newspaper articles [26]. More importantly, they found that media content often presented bottle feeding as easier and as more common than breastfeeding, which was depicted as difficult and more prevalent among the upper middle class and celebrities [26]. In a different part of the world, Henderson determined that discourse in Australian media content both promoted breastfeeding as the best infant feeding choice, while at the same time often suggested that breastfeeding is difficult and not conducive to contemporary lifestyles [27]. Studies also have suggested that media content can play a positive role in shaping infant feeding decisions. Research performed by Arora and colleagues indicated that roughly 90 percent of surveyed mothers who breastfed reported that books, magazines, and television positively influenced their decisions to breastfeed [21].

The purpose of this study is to examine if a relationship between media content on infant feeding and breastfeeding rates exists. We present here results from a content analysis of the advertisements and articles in *Parents' Magazine*, a popular women's publication, which has offered information on health and child development since its inception in 1926 and explore whether those results predict breastfeeding outcomes (for the three decades of annual data available, as discussed below) [28].

We predict the promotion of infant formula usage will negatively affect the prevalence of breastfeeding. Conversely, breastfeeding support in the media should correlate with an increase in breastfeeding rates. More specifically, the following hypotheses guided this study.

### Hypothesis 1 (H1)

The frequency of hand feeding *advertisements *in *Parents' Magazine *from 1971 to 1999 will negatively correlate with changes in breastfeeding reported the following year.

### Hypothesis 2 (H2)

The frequency of *articles *promoting hand feeding in *Parents' Magazine *from 1971 to 1999 will negatively correlate with subsequent changes in reported breastfeeding.

### Hypothesis 3 (H3)

The frequency of articles supporting breastfeeding in *Parents' Magazine *from 1971 to 1999 should positively correlate with subsequent changes in reported breastfeeding.

We focus here on the role played by hand feeding advertisements and articles promoting either hand feeding or breastfeeding. Wolf discusses how the term "hand-feeding," meaning the provision of alternative food to breast milk to infants, was used by middle-class mothers as early as the late 1800s [15]. We continue the usage here, though acknowledge that debate exists as to the most appropriate phrase. Our primary focus on hand feeding advertisements and decision not to also assess the potential impact of advertisements that explicitly promote breastfeeding were driven by the expectation that very few of the advertisements placed in parenting magazines during this time period would promote breastfeeding. (Analysis using the methods described below confirmed this expectation, as no breastfeeding advertisements were found).

## Method

We conducted a content analysis of the advertisements and articles about infant feeding in *Parents' Magazine*, as described below. As Neuendorf notes, content analysis enables a "systematic, objective, quantitative analysis of message characteristics" [29]. For this study, content analysis provided an effective means of classifying and tabulating a large number of advertisements and articles. We then attempted to use that data to predict another time series: annual percentage changes in breastfeeding initiation among U.S. women.

To measure the prevalence of infant feeding advertisements and articles and their change over time, we took a systematic probability sample of content. Specifically, advertisements and articles from the first, fifth, and ninth issues of each year of *Parents' Magazine *from 1971 through 1999 were analyzed. This sample allowed us to assess seasonality and provided data for an extended period of time.

*Parents' Magazine *was a prime candidate for study for a number of reasons. First, it has been in existence from 1926 until the present so its content offers potential variance over time. Secondly, as stated in *Magazines for Libraries*, this magazine "has become the industry standard for parenting magazines" [28]. This publication was also chosen because it focuses on health issues directly relevant to our concerns. *Parents' Magazine *also boasts a large circulation, consistently having more than a million subscribers per year in recent years [28]. Because *Parents' Magazine *has long been a popular magazine for women, it is likely that the information in this publication has not only influenced their behavior, but is also generally reflective of other women's magazines. For all of these reasons, we see data from our *Parents' Magazine *analysis as potentially indicative of general media patterns for the years assessed.

From a preliminary survey of infant feeding advertisements, we created a set of guidelines for the study. All advertisements appearing in the selected issues were considered for analysis. For the purposes of this study, an advertisement is defined as sponsored image or text appearing in the magazine specifically for the purpose of selling a product or promoting a specific behavior. It was then determined if an advertisement pertained to infant feeding, as indicated by text or visual images suggesting that a product was either a food given to infants or a tool used to provide food for infants. "Hand feeding" advertisements included infant formula, cereal/solid food, or hand feeding equipment. Advertisements for cereal and other soft infant foods were included because studies have shown that the introduction of complementary/supplementary foods leads to a decline in breastfeeding duration [30]. With these considerations, advertisements of solid food are included because, like infant formula advertisements, they discourage exclusive breastfeeding and likely discourage breastfeeding initiation, as they suggest other nutritional sources will soon be available for their child. Appendix A provides further detail.

Articles related to infant feeding were also reviewed. To determine if an article pertained to infant feeding, the table of contents of each issue in the sample was reviewed for signifiers of an infant feeding article such as, "bottle, breast, feeding, baby, infant, new mother." If the table of contents indicated a possible feeding article, then the text of the articles, along with images were examined to discover if they discussed hand or breastfeeding. In addition to articles marked as relevant in the table of contents, health question and answer articles were also reviewed (for example, the "Ask the Expert" series). These were included since it is likely that readers receive health information from these pages, therefore may consult them for advice on infant feeding. Articles were considered relevant to infant feeding if any text or images mentioned providing specific nourishment to a baby, either by breastfeeding, hand feeding or either to be considered for analysis. Relevant articles were then classified into one of three categories: "Hand feeding," "Breastfeeding" or "Either". (See Appendix B for the guidelines of article classification).

In order to analyze the relationship between the quantity and type of infant feeding media content and breastfeeding, we needed a consistent measure of national breastfeeding rates. While the National Surveys of Family Growth offers breastfeeding rate information dating back to the 1950s, this survey does not provide annual data. Therefore, we chose the Ross Laboratories Mothers Survey (RLMS) for this study because it contains annual breastfeeding initiation rates, consistently available from 1971 through the present [14,24]. This survey also is commonly cited by other scholars in their discussion of breastfeeding rates in the United States [13-15].

Two coders assessed a sample of advertisements (n = 147) and a sample of articles (n = 10) for the presence of a hand feeding focus, presence of a breastfeeding focus, or lack of relevance to either behavior. Coders agreed perfectly for all variables for both samples. In other words, the two coders achieved a Scott's Pi of 1.0 for all key variables [31].

Southwell and colleagues note that behavior does not usually instantaneously coincide with media exposure, which means that we should include a time lag in attempting to correlate streams of media coverage and reported behavior [5]. Therefore, we correlated breastfeeding data with advertisements and article information from the preceding year. For example, the change in breastfeeding rate reported in 1972 was predicted as a function of the number of relevant advertisements and articles appearing in *Parents' Magazine *in sampled 1971 issues. Using this approach, we assessed an ordinary least squares regression to predict change in breastfeeding rate that included advertisement and article counts from the previous year as independent variables [32].

## Results

**Figure 1:**
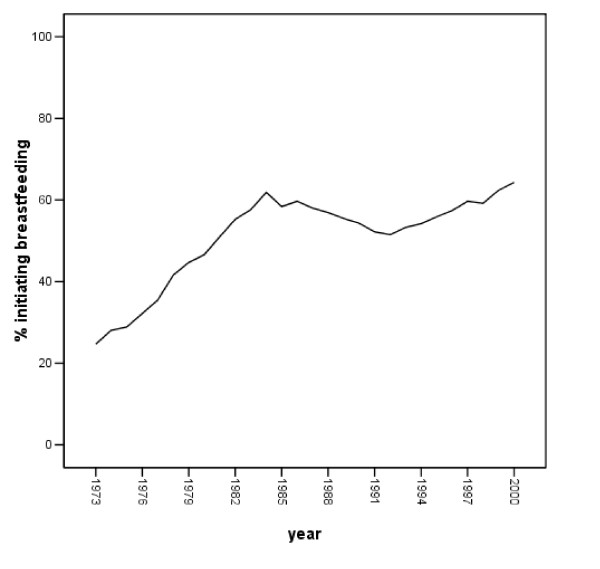
**Percentage of U.S. women initiating breastfeeding, 1973 – 2000**. Note. Breastfeeding data provided by Ross Products Division, Abbott Laboratories [24].

**Figure 2:**
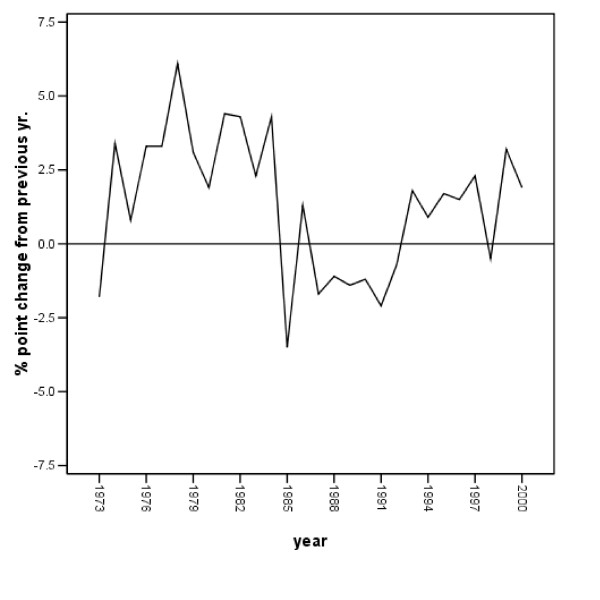
**Change in percentage of women initiating breastfeeding, 1973 – 2000**. Note. Breastfeeding data provided by Ross Products Division, Abbott Laboratories [24].

As noted earlier, breastfeeding did rise substantially over the period investigated. Over the nearly three decades assessed, the proportion of women initiating breastfeeding rose from approximately one-quarter to 64 percent in 2000 [14,24]. Figure 1 illustrates these changes. Figure 2, however, also indicates that there was variation in the annual change documented; it is that change we analyze here.

**Figure 3:**
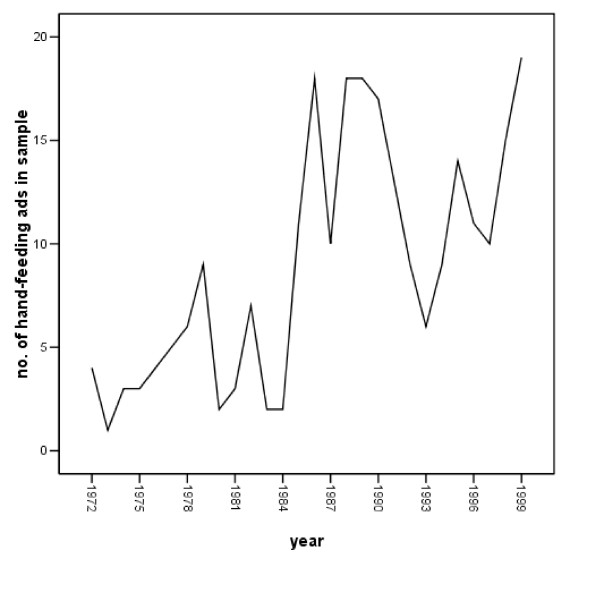
Number of hand-feeding advertisements in magazine sample, 1972 – 1999.

At the same time, there apparently were a substantial number of advertisements for hand-feeding products during parts of this period. A large number of advertisements supporting hand feeding appeared in our *Parents' Magazine *sample; 249 advertisements involved hand feeding in the 84 issues analyzed. Figure 3 illustrates the frequency of such advertisements across 28 years.

Most of these advertisements were classified as "Cereal/Solid" food. Due to the scarcity of infant formula advertisements, and the similarity between these advertisements and the ones featuring cereal and solid food, "infant formula," "cereal/solid food" and "hand feeding equipment" were analyzed together under the larger classification of "hand feeding" advertisements. Further, no advertisements for the general promotion of breastfeeding or for breastfeeding products appeared in the sample.

**Figure 4:**
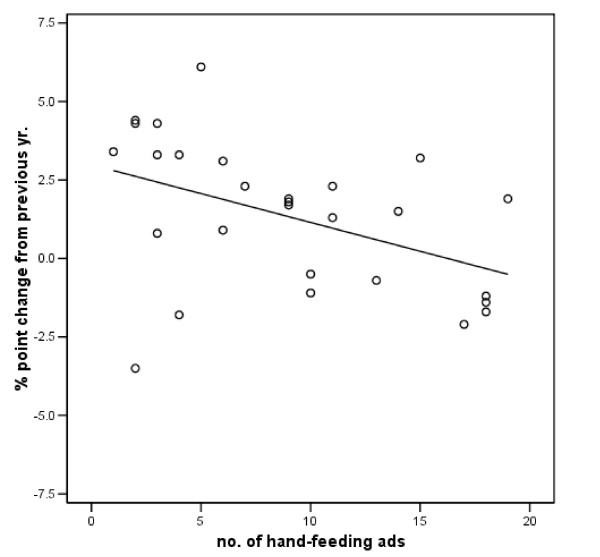
Relationship of number of hand-feeding advertisements and subsequent changes in reported breastfeeding.

Evidence supported hypothesis one, which predicted a negative relationship between the number of hand feeding advertisements and change in breastfeeding rates. Table 1 provides the relevant coefficients. As expected, when the frequency of hand feeding advertisements increased, the percentage change in breastfeeding rates reported the next year tended to decrease, p < .05. Figure 4 provides a graphical illustration of this relationship.

**Table 1:** Regression results for prediction of annual percent change in breastfeeding, 1973–2000

Explanatory variable	B	SEB
Number of hand feeding ads (prior year)	-.20*	.07
Number of hand feeding articles (prior year)	.35	.71
Number of breastfeeding articles (prior year)	.61	.41

Note. Breastfeeding initiation figures come from the Ross Laboratories Mothers Survey [24] and were entered with a one-year time lag relative to advertisements. For example, advertising in 1972 was used to predict a change in breastfeeding initiation that was measured in the 1973 annual study. In the table, B refers to the unstandardized regression coefficient. R^2 ^for the model was .27 and n = 28 (years). * p < .05.

Hypothesis two predicted a negative relationship between the frequency of hand feeding articles and changes in breastfeeding levels. Our evidence from the reported regression model does not support this contention. We also expected that a positive correlation would exist between the frequency of breastfeeding articles and changes in subsequent breastfeeding levels (H3). The results for hypothesis three are directionally consistent with our prediction, though the coefficient for breastfeeding articles was not statistically significant in the final model.

## Discussion

Results indicate that there is a relationship between quantity of some types of media content and health behavior. Specifically, the frequency of hand feeding advertisements negatively predicted breastfeeding in new mothers. This finding is important because it suggests that hand feeding advertisements are negatively connected with breastfeeding rates, which, in turn, has several implications. First, this negative correlation may help explain why historically, breastfeeding rates were higher in some decades than others, despite similar external forces that should have positively affected breastfeeding. Fluctuations in these types of advertisements in this magazine as well as others may have impacted women's breastfeeding decisions in ways unaccounted for in many contemporary historical accounts.

Besides providing insight into underlying motivations behind infant feeding in the past, these results also have implications for the future. Since these advertisements continue to be presented to American women and others around the globe, it is likely that hand feeding media content still discourages women from breastfeeding, despite the fact that most physicians generally adhere to the belief that breastfeeding provides the healthiest start for an infant [19]. (The overall absence of breastfeeding advertisements, then, also is notable because it means that no equivalent advertising counter-message to the hand feeding advertisements existed in this forum).

Several limitations to this study exist. It is important to note that we do not have direct evidence of causation. Many factors likely influence any one woman's decision to breastfeed. Our intention was to demonstrate a statistical relationship between advertising and breastfeeding rates and we have done that. What we need next is evidence regarding the exact mechanism for this relationship.

It is important to keep in mind that this study focused on three decades in the United States, a time and a place that may or may not mirror other locations around the globe or other time periods in terms of advertising laws and norms. In fact, there has been considerable discussion and debate over whether and how to limit the marketing of substitutes for, and alternatives to, breastfeeding [33]. The impact of advertising found here might be generalizable only to the last part of the 20^th ^century in the United States.

We also found no evidence for a significant relationship between either hand feeding or breastfeeding articles and changes in breastfeeding rates. One possible explanation of why this relationship was not present, however, is that *Parents' Magazine *may not be fully characteristic of all news coverage. This magazine may better represent fluctuations in national levels of advertising related to infant feeding in print media during these years than it represents rises and falls in articles on this topic. A future study could examine additional indicators of this news coverage, such as more years of issues with infant feeding articles or other media.

The direction of the observed (but non-significant) relationship between our count of breastfeeding articles and breastfeeding rates nonetheless is suggestive, if not conclusive. Here limited data and time points constrain our discussion. In general, though, analyses suggest that hand feeding articles most commonly appeared before the mid-1970s, when breastfeeding rates were low. Breastfeeding articles, in contrast, dominated the second half of the 1970s, which does correspond with rising breastfeeding rates, as well as the 1980s and 1990s, when it was rare to find a hand feeding article. Most articles promoted either bottle or breastfeeding as viable options or exclusively encouraged breastfeeding. Most articles from the last thirty years, overall, promoted breastfeeding and, generally speaking, breastfeeding has increased since the early 1970s.

Our work suggests numerous directions for further study. One obvious avenue would entail finding additional data with regard to breastfeeding that would permit a longer time-series for study, as we know there is variance in both media content and breastfeeding in the years prior to the 1970s. Certainly, additional time points would boost our power to detect effects. In addition to our discussion about general trends in breastfeeding in the early and mid-20^th ^century, a preliminary survey of the hand feeding advertisements in years prior to the decades studied here (for which annual breastfeeding data was not readily available), for example, suggests a significant change in approach from the 1950s through the 1990s.

A qualitative or more in-depth analysis of such advertisements and articles also may help explain not just how the frequency of content varied, but how the messages themselves changed over time. In addition, this study was limited to *Parents' Magazine*, as we have noted. It could be that other media content could offer the potential for broader generalizations about the relationship between the media and infant feeding decisions.

## Conclusion

We endeavored here to examine the relationship between media content and health behavior, specifically exploring the potential role of popular culture products in influencing infant feeding decisions. We predicted that the frequency of advertisements and articles featuring hand feeding would be negatively correlated with changes in breastfeeding and also predicted that a positive relationship would exist between advertisements and articles promoting breastfeeding and changes in breastfeeding rates.

As predicted, hand feeding advertisements were negatively correlated with changes in breastfeeding rates. When hand feeding advertisements rose, subsequent reports of breastfeeding generally fell. Results were less supportive with regard to articles: while some patterns observed were directionally consistent with predictions, neither article coefficient was statistically significant.

These results bear important implications. The trends in the frequency of hand feeding advertisements seem to statistically predict variations in breastfeeding rates in the United States, which is consistent with the contention that media content played a role in many infant feeding decisions. This data also suggests that advertising in magazines and related media outlets may have served as a common thread with regard to infant feeding rate changes in the late 20th century. Such advertising may have strengthened and perpetuated ideologies against breastfeeding, which may explain why breastfeeding rates fluctuated even when many of the previously documented reinforcing factors remained the same.

Since these results indicate that there is a relationship between media messages and breastfeeding rates, some might argue popular culture products should aim to support breastfeeding. Physicians generally agree that breastfeeding is the best nourishment for infants; therefore, it is crucial that media efforts support this infant feeding choice in the face of counter-persuasive efforts on the part of commercial entities promoting hand feeding.

## Competing interests

The author(s) declare that they have no competing interests.

## Authors' contributions

Both authors contributed equally to this work.

## Appendix A – guidelines for classifying "hand feeding" advertisements

An advertisement was considered relevant to hand feeding if the text or images suggested that the featured product offered an alternative to breastfeeding. In light of that, relevant advertisements included three types of hand feeding products: formula, cereal/solid food, or hand feeding equipment.

In "formula" advertisements, the product is specifically designed for infants, as indicated by text, e.g., "Great infant food!" or by images, e.g., a picture of an infant, and the advertisement denotes that the product is designed to be consumed as a liquid, as indicated by text, e.g., "milk substitute" or images, e.g., picture of an infant's bottle. For this study, advertisements for milk substitutes, condensed milk, powdered milk were included if the text or images demonstrate that they are to be used for infants. Advertisements that were excluded from the study featured products designed to boost caloric intake or alter flavor of existing drink or to increase nutrients (e.g., Cocoamalt, Karo, Yeast Foam tablets, malted milk, Ovaltine).

In "cereal/solid food" ads, the featured product is a food marketed towards infants, as indicated by text, e.g., "For infants six months and older" or images, e.g., an infant eating the product and if text indicates that product is a hot cereal (Farina cereal, for example) or solid food designed for babies, e.g., strained peas for infants.

For this study, hot cereals repeatedly marketed toward the feeding of infants such as Farina, Wheatena, Cream of Wheat, and KinderGruel were included, as well as vegetables, soup, and meat specifically designed for infants. Oatmeal and other hot cereal, like Heinz breakfast, Raston, and Postum were only included if the advertisement specifically addresses infants through text or images of infants. If no mention of infants appears in text or images of the ad, then the hot cereal advertisement for the products listed above was excluded. Advertisements for food products, which do not specifically indicate that they are intended for infants, e.g., many pudding ads and advertisements directed at older children were excluded from this study.

In "hand/bottle feeding equipment" ads, the featured product was designed to directly assist in hand/bottle feeding an infant as indicated by text or images, e.g., "Bottle sterilizer". Products used in hand feeding or to assist hand feeding, such as bottles, nipples, bottle warmers, sterilizers, and holders; and bottle top closures (like a welded wire seal) were included in this study. Products indirectly connected to hand/bottle feeding, and products, which may be used for either bottle or breastfeeding (e.g. food strainer, infant clothing) were excluded from the study, as were products explicitly labeled as assistants for breastfeeding, e.g., bottles for feeding pumped breast milk.

## Appendix B – guidelines for classifying infant feeding articles

To be considered a "hand feeding" article, the text within the article must have contained at least one statement indicating formula (or breast milk substitutes) as either the only option available to feed infants, the best option to feed infants or the most realistic option to feed infants, e.g., breastfeeding might be superior, but it is too difficult. An article also counted as "hand feeding" if the article itself centered on infant feeding (indicated by the headline and lead paragraphs) and contains an image of an infant with a bottle.

A "breastfeeding" article was characterized by text within the article, which contained at least one statement indicating that breastfeeding is either the only option available to feed infants or the best option to feed infants. An article was also considered a "breastfeeding" article if the text centers on infant feeding (as indicated by the headline and lead paragraphs) and contained an image of an infant breastfeeding.

Articles were classified as "either" if the text or images suggested that both breast and hand feeding were equally suitable infant feeding options, e.g., "breastfeeding is less expensive, yet formula is more convenient". Articles were excluded if neither breast nor hand feeding (or tools for these options) were discussed in the article, e.g., a referral to feeding an infant without specifically referring to breast or bottle feeding.
